# Late onset hyperornithinemia-hyperammonemia-homocitrullinuria syndrome - how web searching by the family solved unexplained unconsciousness: a case report

**DOI:** 10.1186/s13256-018-1794-9

**Published:** 2018-09-23

**Authors:** Thomas Silfverberg, Fredrik Sahlander, Magnus Enlund, Mikael Oscarson, Maria Hårdstedt

**Affiliations:** 1 Department of Clinical Science and Education at Södersjukhuset, Karolinska Institutet, Stockholm, Sweden; 2 Department of Internal Medicine, Falun Hospital, Falun, Sweden; 3 Center for Clinical Research Dalarna-Uppsala University, Falun, Sweden; 4 Department of Intensive Care, Falun Hospital, Falun, Sweden; 5 Department of Molecular Medicine and Surgery, Karolinska Institutet, Stockholm, Sweden; 6 Department of Endocrinology, Metabolism and Diabetes, Karolinska University Hospital, Stockholm, Sweden; 7 Centre for Inherited Metabolic Diseases, Karolinska University Hospital, Stockholm, Sweden; 8 Department of Cardiology, Falun Hospital, Falun, Sweden

**Keywords:** Ammonia, HHH, Hyperammonemia, Hyperornithinemia-hyperammonemia-homocitrullinuria syndrome, UCD, Unconsciousness, Urea cycle disorders

## Abstract

**Background:**

Hyperornithinemia-hyperammonemia-homocitrullinuria syndrome, a rare inherited urea cycle disorder, can remain undiagnosed for decades and suddenly turn into an acute life-threatening state. Adult presentation of hyperornithinemia-hyperammonemia-homocitrullinuria syndrome has rarely been described, but is potentially underdiagnosed in the emergency room. In the case of acute hyperammonemia, prompt diagnosis is essential to minimize the risk of brain damage and death.

**Case presentation:**

We present the diagnostics, clinical course, and treatment of a 48-year-old Caucasian man presenting with unexplained unconsciousness in the emergency room. A web search by a family member led to the suspicion of urea cycle disorder. Subsequent analysis of plasma ammonia and amino acids in plasma and urine demonstrated a pattern typical for hyperornithinemia-hyperammonemia-homocitrullinuria syndrome. The diagnosis was confirmed by genetic analysis which revealed two heterozygous mutations in the *SLC25A15* gene. The cause of the hyperammonemia crisis was acute upper gastrointestinal hemorrhage, leading to protein overload and subsequent cerebral edema. Continuous renal replacement therapy, scavenger treatment, and tightly controlled nutrition were useful in preventing hyperammonemia and recurrence of cerebral edema.

**Conclusions:**

The case emphasizes the importance of taking rare metabolic genetic disorders into consideration in patients with prolonged unexplained unconsciousness.

## Background

Unexplained unconsciousness is a major challenge in emergency medicine. Such patients generally require prompt diagnosis and treatment, as well as continuous monitoring. Metabolic disturbances, for example, diabetic coma, hepatic or renal failure, hypothyroidism, and severe electrolyte disturbances, can cause unconsciousness but are generally not missed owing to routinely available tests. However, rare inherited metabolic disorders, such as urea cycle disorders (UCDs), can present with unexplained unconsciousness and be difficult to identify.

The incidence of UCDs is estimated to be 1:35,000 live births. Hyperornithinemia-hyperammonemia-homocitrullinuria (HHH) syndrome is thought to account for 1–3.8% of all UCDs [1–6]. UCDs are most often diagnosed during the first years of life. Approximately 100 cases of HHH syndrome have been reported in the literature since the disorder was first described in the 1960s. Approximately 90% of the patients with HHH syndrome develop symptomatic disease as a neonate, infant, or child. The diagnosis is often delayed, with a mean diagnostic delay of approximately 6 years. One third of the patients are diagnosed over 12 years of age [1].

The urea cycle is a metabolic pathway that occurs in the liver and involves both the mitochondria and the cytosol, with entry of ornithine into the mitochondria in exchange for citrulline. This cycle converts the highly toxic ammonia to urea, which is less toxic, more soluble, and easily excreted into the urine. Enzyme dysfunction at different steps of the urea cycle can lead to accumulation of ammonia. Although UCDs are usually diagnosed in early childhood, partial enzyme deficiencies can remain silent until rare episodes of protein overload and/or increased catabolism with metabolic decompensation result in intercurrent hyperammonemia. The risk of hyperammonemia is increased by prolonged fasting, surgery, trauma, pregnancy, and episodes of increased catabolism, for example in infections such as gastroenteritis [7–9].

HHH syndrome (Online Mendelian Inheritance in Man (OMIM) 238970) belongs to the group of UCDs. HHH syndrome is a rare autosomal recessive disorder caused by mutations in the *SLC25A15* gene, causing reduced or abolished activity of the mitochondrial ornithine carrier ORC1 [10, 11]. ORC1 is expressed in peripheral tissues and periportal hepatocytes. It transports ornithine, lysine, and arginine from the cytosol to the mitochondrial matrix where urea synthesis takes place [12]. Reduced activity of ORC1 leads to accumulation of ornithine in the cytosol and subsequent *hyperornithinemia*. The lack of mitochondrial ornithine results in reduced ornithine transcarbamylase (OTC) activity, which slows down the urea cycle, leading to accumulation of carbamoyl-phosphate and *hyperammonemia.* Carbamoyl-phosphate either forms orotic acid through the pyrimidine pathway or binds with lysine to produce homocitrulline explaining the *homocitrullinuria*. Ammonia is toxic to the brain in high concentrations. When plasma ammonia rises rapidly, it penetrates the blood–brain barrier and is converted to glutamine in astrocytes, which attracts water through osmosis and disruption of aquaporin channels causing cerebral edema. The electrolyte homeostasis of the brain is disrupted which adds to the toxic effect [13].

The acute presentation of HHH syndrome, as well as other UCDs, includes neurological symptoms (altered level of consciousness, dysphasia, abnormal motor function, drop attacks, seizures, gait disturbances, and behavioral changes), gastrointestinal symptoms (vomiting, loss of appetite, nausea, diarrhea, and constipation), liver failure, respiratory alkalosis, and coagulation disturbances [1–3]. Chronic presentation of HHH syndrome includes aversion for protein-rich foods and progressive encephalopathy including motor dysfunction, mental retardation, and intellectual disability [1, 3]. HHH syndrome responds well to long-term treatment (low protein diet supplemented with citrulline and/or arginine, combined with ammonia scavengers) and relapse of acute hyperammonemia is rare [13]. However, chronic disease can result in a pyramidal syndrome with progression to spastic paraparesis [4, 14].

Metabolic decompensation and intercurrent hyperammonemia can occur at any time in patients with UCDs, resulting in critical illness. The duration and severity of hyperammonemia correlates with brain damage [6]. Therefore, early diagnosis and treatment is essential to improve the outcome.

## Case presentation

A 48-year-old Caucasian man was admitted to our emergency ward. His medical history included an episode of depression and some disabilities in reading and writing. From the age of 1 to 8 years, he had recurrent episodes of seizures associated with vomiting and loss of unconsciousness, which had been interpreted as febrile seizures. Ten years earlier he was diagnosed as having a peptic ulcer of the stomach after episodes of modest gastrointestinal bleeding. He had independently excluded protein from his diet during several periods of his life.

He presented at the emergency room (ER) at 8 a.m. with complaints of headache, backache, and a sensation of pressure in his ears since the previous evening. He had felt nauseous and vomited several times during the night. Late in the night he developed altered sensorium and his wife brought him to the hospital. In the ER, he initially answered monosyllabically to questions (Glasgow Coma Scale (GCS) 11) but within 15 minutes he lost consciousness (GSC 8). He was afebrile and his circulatory/respiratory systems were stable. On neurological examination he was motorically agitated, moved all extremities, and presented slight miosis and saccadic eye movements. The Babinski sign was positive bilaterally. Routine hematological and biochemical blood tests, including blood cell counts, electrolytes, liver parameters, intoxication screening, glucose, and C-reactive protein (CRP), turned out normal. Arterial blood gases showed a lactate concentration of 2.1 mmol/L and a pH of 7.5. A computed tomography (CT) scan of his brain was normal.

During the *12 hours* after admission, he was still unconscious but could breathe autonomously and was circulatory stable. A lumbar puncture revealed a slight rise in lactate (3.7 mmol/L), but no signs of infection or inflammation. An electroencephalogram (EEG) showed suspected encephalopathy with pronounced pathological activity but no focality, asymmetry, or epileptic activity. Broad-spectrum antibiotic and antiviral treatment was given despite no obvious signs of central nervous system (CNS) infection. Twelve hours after admission, he had hematemesis and due to respiratory instability he was transferred to our intensive care unit (ICU), anesthetized, and intubated. Concerns regarding a possible inflammatory lesion in his CNS led to administration of betamethasone. Acute magnetic resonance imaging (MRI), including in-flow angiography, phase-contrast angiography, and diffusion series, was normal.

The following morning, *24 hours* after admission, the cause of unconsciousness was still unclear. The contribution by the relatives of our patient here became essential. His sister called for attention regarding her suspicion that her brother might suffer from a UCD. She explained that the relatives had discussed the possibility that her brother might be suffering from a congenital neurological disorder based on his seizures, cognitive difficulties, and dyslexia since childhood. Because of the acute severe illness, they made a search on a web search engine using a combination of his symptoms, which was vomiting, unconsciousness, and seizures (in Swedish, *kräkningar*, *medvetslöshet*, *kramper*). The web search resulted in the suspicion of OTC deficiency, which belongs to the group of UCDs. Our patient’s plasma ammonia concentration was therefore analyzed and turned out to be 213 μmol/L (reference level 11–32 μmol/L), diagnostic for a UCD in the absence of hepatic failure. Continuous renal replacement therapy (CRRT) was initiated to eliminate plasma ammonia.

**Fig. 1:**
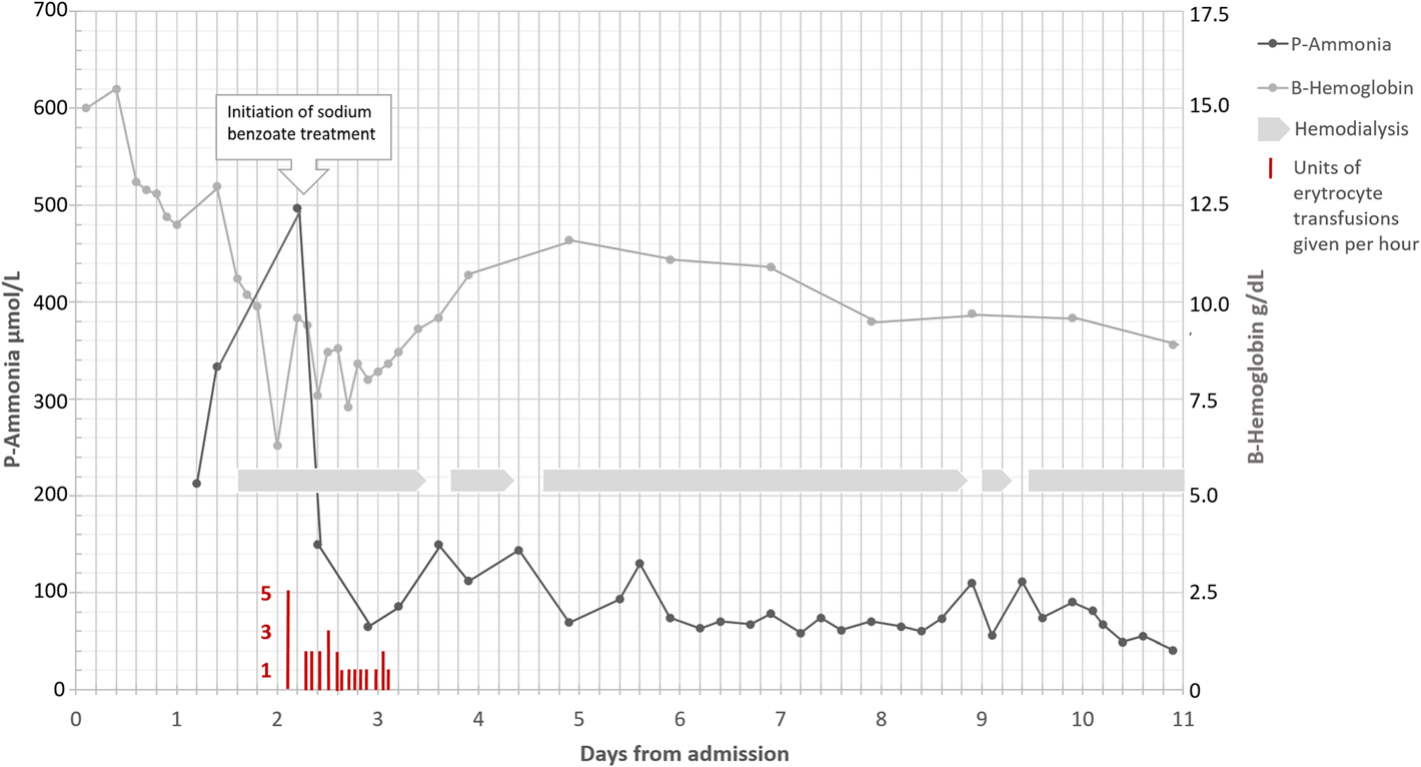
Ammonia (μmol/L) and hemoglobin (g/dL) levels in a 48-year-old man with hyperornithinemia-hyperammonemia-homocitrullinuria syndrome during an acute episode of hyperammonemia. Days from admission are presented on the *x*-axis. The diagnosis was suspected on day 2 and active treatment started. Initiation of treatment with scavenger (sodium benzoate) and duration of continuous renal replacement therapy is illustrated as well as erythrocyte transfusions

Approximately *48 hours* after admission, our patient became circulatory unstable. Acute gastroscopy revealed a severe upper gastrointestinal hemorrhage from a peptic ulcer of his stomach. The ulcer was treated locally through gastroscopy and he needed repeated erythrocyte and plasma transfusions (Fig. 1). Heparin was suspended during CRRT. Despite this treatment, his ammonia rose to 497 μmol/L and he developed recurrent partial seizures (Fig. 1). Sodium benzoate, an ammonia scavenger, was given as a bolus infusion of 250 mg/kg for 120 minutes, followed by continuous maintenance infusion of 250 mg/kg per day with addition of carnitine and arginine substitution. During the following 30 hours, he suffered from recurrent gastric bleedings from his peptic ulcer which were repeatedly treated locally through gastroscopy. He received a total of 25 units of erythrocytes, 20 units of plasma, and 3 units of thrombocytes, in addition to intravenously administered fluids. The total volume of blood lost through bleeding was estimated to be 10 liters.

On *day 3*, biochemical analysis confirmed the diagnosis of HHH syndrome. In addition to elevated levels of ammonia, the plasma concentration of ornithine was 420 μmol/L (reference 30–110 μmol/L) and homocitrulline in urine was detected (23 mmol/mol creatinine; normally not detected). Further analysis of plasma amino acids also showed elevated glutamine (1321 μmol/L; reference 355–725 μmol/L), whereas citrulline (30 μmol/L; reference 15–50 μmol/L), arginine (33 μmol/L; reference 30–125 μmol/L), and lysine (221 μmol/L; reference 20–221 μmol/L) were in the normal range. Orotic acid in urine was 156 mmol/mol creatinine (reference < 1.0 mmol/mol creatinine). The concentrations of several essential amino acids were low.

Intravenously administered glucose and lipids were given to keep a preset blood glucose level, in order to stimulate insulin secretion and prevent catabolism. Total nutrition level was aimed at 115% of normal needs and no proteins were given. At the end of the third day his ammonia level was in the normal range.

**Fig. 2:**
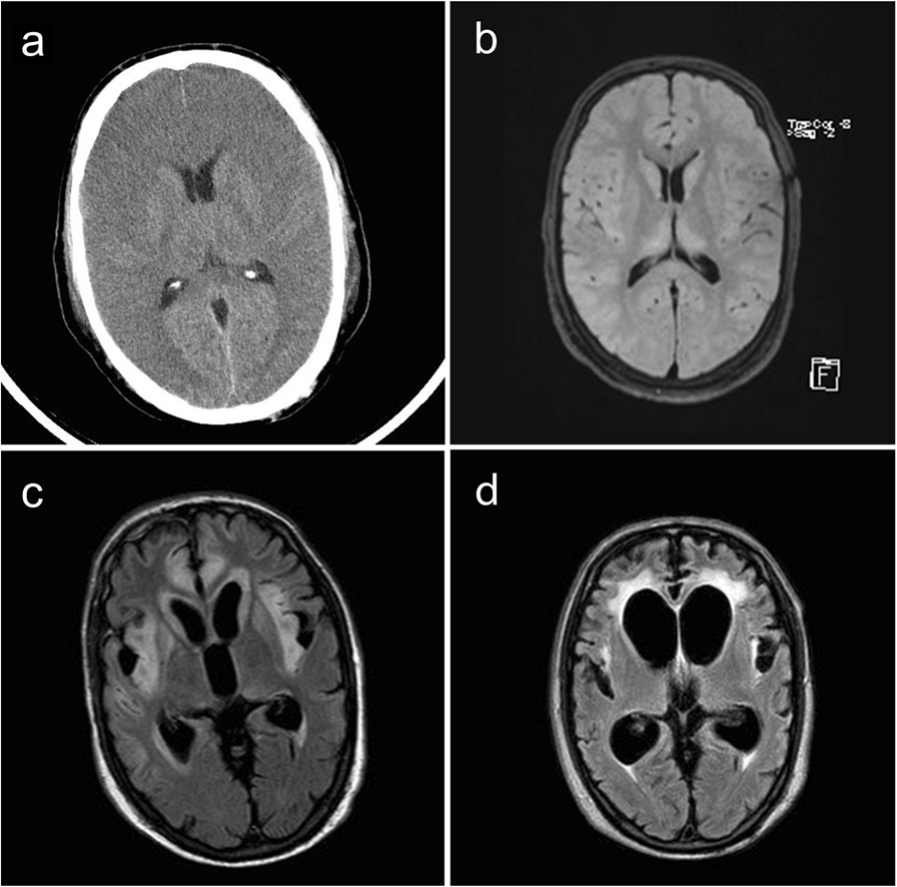
Cranial imaging at different time points from a 48-year-old man with acute hyperammonemia and hyperornithinemia-hyperammonemia-homocitrullinuria syndrome. Computed tomography of the brain 4 days after admission shows a considerable general cerebral edema (**a**). Magnetic resonance imaging at day 5 shows a widespread cytotoxic edema in insula, gyrus cingula, and the temporal and frontal lobes (**b**). Magnetic resonance imaging at day 31 demonstrates regression of edema, widespread gliosis in the frontotemporal lobes bilaterally, and hemorrhagic necrosis/malacia in frontotemporal cortex and basal ganglia bilaterally (**c**). Magnetic resonance imaging 7 months after admission shows pronounced progress of gliosis development, widespread malacia in the tips of the temporal lobes with greatly widened temporal horns of the lateral ventricles (**d**)

Late on *day 4*, a wake-up call was attended, which revealed deep unconsciousness with stereotypic extension of limbs. He was again anesthetized and a CT scan of his brain showed considerable, general cerebral edema (Fig. 2a). He was transported to a neurological ICU. Continuous monitoring showed increased intracranial pressure (ICP) and he was treated with deeper sedation. He developed epileptic convulsions and was given levetiracetam. MRI on day 5 showed generalized cytotoxic edema in cortex and subcortical white matter; the edema was symmetric and more prominent in insula, gyrus cingula, and hippocampus compared to the basal ganglia and infratentorially (Fig. 2b). This pattern is considered typical for hyperammonemic encephalopathy [15]. The ICP gradually decreased. CRRT was discontinued on *day 15* and the ammonia concentration remained normal. He was treated with protein-reduced food, sodium benzoate, arginine, citrulline, and substitution of essential amino acids. On *day 21* he was transferred back to the general ICU, where he was treated for another 9 days. MRI of his brain on *day 31* showed regression of edema (Fig. 2c).

He remained hospitalized for another 9 months, mainly for rehabilitation. He suffered from considerable impressive and expressive aphasia (monosyllabic), perception difficulties (visual, auditory, and spatial), spastic paraparesis, and dystonia. He could move his head autonomously, but could not control his arms and legs. Despite a few bacterial infections, aspiration pneumonia, and urine tract infections, no hyperammonemia was observed. MRI of his brain after 7 months showed considerable atrophy, general gliosis, and wide ventricles (Fig. 2d). During the rehabilitation the severity of the spastic paraparesis was progressive.

*Ten months* following admission he was discharged from our hospital after his home had been adapted to his needs. The treatment with ammonia scavengers (sodium benzoate) was continued as well as substitution with citrulline, arginine, and essential amino acids. His protein intake was reduced to a level of 0.7 (0.6–0.8) g/kg per day, in accordance to minimum protein intake for adult male based on World Health Organization (WHO) guidelines [3]. His ammonia concentration has remained within normal ranges or discretely elevated at times of acute illness (an episode of gastroenteritis and urinary tract infection).

*Fifteen months* after his first admission to our hospital, he has started to eat unassisted and use his legs to move his wheelchair around in his apartment. He has also begun to use more words in his speech (still monosyllabically).

Sequence analysis of the *SLC25A15* gene (Center for Genomics and Transcriptomics, Tübingen, Germany) detected two heterozygous mutations: a c.337G>A (p.G113S) mutation in exon 4 and a c.712C>T (p.Q238X) mutation in exon 6. Our patient’s mother and daughter were heterozygous carriers only for the c.712C>T mutation whereas his sister was a heterozygous carrier of the c.337G>A mutation confirming that our patient carried the two mutations in trans. The c.712C>T mutation in exon 6 leads to a premature stop codon and thus a truncated and probably inactive protein. The mutation c.337G>A (p.G113S) in exon 4 is listed in the Single Nucleotide Polymorphism Database (dbSNP; rs199894905) with an allele frequency of 0.1%. This missense mutation affects a phylogenetically highly conserved amino acid residue and *in silico* analysis with different prediction programs (for example, PolyPhen-2 and SIFT) classifies the mutation as pathogenic. Another mutation affecting the same residue (p.G113C) has also been described in another patient with HHH [16].

## Discussion and conclusions

This case report acknowledges the importance of recalling rare metabolic diseases, such as UCDs, in prolonged unexplained unconsciousness in adults. Since an acute episode of hyperammonemia can result in permanent brain damage, lifelong disabilities, and have a potentially fatal outcome, a correct diagnosis and timely treatment is essential. Our case highlights the importance of paying attention to information given by relatives and friends. It also shows the power and efficiency of modern web search engines as useful tools when facing rare disorders with heterogeneous presentation and several different symptoms. Our patient may not have survived, if the diagnosis suggested by the family had not been considered and our patient investigated along those lines.

Hyperammonemia, hyperornithinemia, and urinary excretion of homocitrulline is the metabolic triad defining HHH syndrome. These characteristic laboratory abnormalities were all confirmed for our patient in the acute stage. The diagnosis was later supported by a genetic analysis which showed that he was compound heterozygous for two mutations in *SLC25A15* gene. One mutation (c.712C>T; p.Q238X) results in a truncated and probably inactive transporter, whereas the second mutation (c.337G>A; p.G113S) probably results in a transporter with reduced activity. Our patient’s phenotype with limited clinical symptoms throughout life, until the gastrointestinal hemorrhage caused an endogenous protein overload and subsequent metabolic decompensation, is also consistent with an ORC1 transporter with some residual activity.

The acute clinical course of our patient was complicated by upper gastrointestinal bleeding, requiring blood and plasma transfusions that resulted in protein overload and subsequent cerebral edema. Elimination of plasma ammonia by CRRT and scavenger treatment, together with prevention of the acute catabolic state became critical parts of the initial intensive care management [17]. As demonstrated in Fig. 1, plasma ammonia declined after initiation of the scavenger treatment even though the gastrointestinal hemorrhage persisted. CRRT most probably also contributed to the decrease in ammonia plasma concentration. When CRRT was discontinued at later points, plasma ammonia concentration increased, suggesting the effectiveness of dialysis treatment (Fig. 1). The therapeutic nutritional treatment diverged substantially from the regular nutritional principles by higher energy content, initially with complete exclusion of proteins. Proteins were introduced with caution and tight monitoring of ammonia concentration. As his condition became more critical, continuous monitoring of ICP was required and he was transferred to a neurological ICU. At 15-month follow-up, the protein-reduced diet and treatment with scavengers had been sufficient to prevent hyperammonemia.

Although HHH syndrome is rarely diagnosed in adults, it might be more frequent than currently reported due to difficulties in early diagnosis. Our interpretation of the present case is that patients with *SLC25A15* gene mutation(s), resulting in a partial functioning ORC1 transporter, can live a life with very limited symptoms until an acute situation of relative protein overload tips them over the edge, resulting in potentially fatal hyperammonemia. In summary, we emphasize the recommendation of European UCD guidelines to measure ammonia in patients presenting with prolonged unexplained unconsciousness, irrespective of age [3].
